# Minds “At Attention”: Mindfulness Training Curbs Attentional Lapses in Military Cohorts

**DOI:** 10.1371/journal.pone.0116889

**Published:** 2015-02-11

**Authors:** Amishi P. Jha, Alexandra B. Morrison, Justin Dainer-Best, Suzanne Parker, Nina Rostrup, Elizabeth A. Stanley

**Affiliations:** 1 Department of Psychology, University of Miami, Miami, Florida, United States of America; 2 Walsh School of Foreign Service and Department of Government, Georgetown University, Washington, District of Columbia, United States of America; 3 Mind Fitness Training Institute, Alexandria, Virginia, United States of America; Centre de Neuroscience Cognitive, FRANCE

## Abstract

We investigated the impact of mindfulness training (MT) on attentional performance lapses associated with task-unrelated thought (i.e., mind wandering). Periods of persistent and intensive demands may compromise attention and increase off-task thinking. Here, we investigated if MT may mitigate these deleterious effects and promote cognitive resilience in military cohorts enduring a high-demand interval of predeployment training. To better understand which aspects of MT programs are most beneficial, three military cohorts were examined. Two of the three groups were provided MT. One group received an 8-hour, 8-week variant of Mindfulness-based Mind Fitness Training (MMFT) emphasizing engagement in training exercises (training-focused MT, *n* = 40), a second group received a didactic-focused variant emphasizing content regarding stress and resilience (didactic-focused MT, *n* = 40), and the third group served as a no-training control (NTC, n = 24). Sustained Attention to Response Task (SART) performance was indexed in all military groups and a no-training civilian group (CIV, *n* = 45) before (T1) and after (T2) the MT course period. Attentional performance (measured by A’, a sensitivity index) was lower in NTC vs. CIV at T2, suggesting that performance suffers after enduring a high-demand predeployment interval relative to a similar time period of civilian life. Yet, there were significantly fewer performance lapses in the military cohorts receiving MT relative to NTC, with training-focused MT outperforming didactic-focused MT at T2. From T1 to T2, A’ degraded in NTC and didactic-focused MT but remained stable in training-focused MT and CIV. In sum, while protracted periods of high-demand military training may increase attentional performance lapses, practice-focused MT programs akin to training-focused MT may bolster attentional performance more than didactic-focused programs. As such, training-focused MT programs should be further examined in cohorts experiencing protracted high-demand intervals.

## Introduction

Soldiers are experts at *standing at attention*, a body posture that conveys motionless alertness. While this expertise is achieved after a few short weeks of basic training, training to promote a *mind ‘at attention’* seems far more elusive. Such a training program would need to have as its primary goal strengthening attention and curbing the mind’s pervasive tendency to fluctuate from the external task environment toward internally generated thoughts, feelings, and preoccupations, which may be unrelated to the task at hand. From this perspective, the antithesis of a mind ‘at attention’ is a wandering mind.

There is growing evidence that mind wandering, described as off-task (vs. on-task) stimulus-independent thinking during ongoing task performance, results in attentional performance lapses [1–5]. Theoretical models suggest that these lapses may be due to the ‘perceptual decoupling’ of attention [6–8]. During off-task episodes, attentional resources necessary for task-related cognitive and perceptual analysis of environmental stimuli are decoupled from the task at hand as attention is commandeered by internally generated thought.

Attentional lapses driven by off-task thinking could have particularly deleterious effects when experienced by individuals whose jobs require situational awareness, surveying environmental input to detect low-probability events or rapidly changing circumstances [9,10]. Further, when attention is derailed in this manner, the ability to monitor and adjust behavior or take corrective action based on real-time feedback can be compromised [11]. As such, attentional lapses could have dire consequences for a variety of professions for whom present-moment attention to immediate environmental input is critical, from air traffic controllers to pilots, police officers, fire fighters and troops on patrol.

Unfortunately, mind wandering episodes are not rare. Experience-sampling studies suggest that off-task thinking happens 30% [12] to nearly 50% [13] of waking hours. Furthermore, self-initiated reports of mind wandering tend to underestimate its occurrence relative to frequencies captured by inclusion of probe-caught methods in experimental paradigms that include both of these methods [14,15]. This underestimation may be because mind wandering, more so than other mental events, is characterized by a lack of meta-awareness, the knowledge one has of his or her own conscious experience at any given moment [6,8].

Given the imperfect nature of subjective reports of mind wandering, recent efforts aim to determine if objective performance measures may better identify its occurrence. Bastian and Sackur [16] investigated if response time (RT) variability significantly correlates with probe-elicited self-reports of off-task thinking during a continuous performance task. They argued that the variability in RT may reflect the waxing and waning of attentional resources engaged in the task at hand. Indeed, they found that higher RT variability corresponded with greater self-reported mind wandering. Next, they went on to determine if RT variability predicted the likelihood of reporting off-task thinking in a real-time fashion during the ongoing task. They found that reports of off-task thinking were more likely in response to probes that were temporally near vs. far from local peaks in RT variability. Based on this, they suggested that RT variability, itself, may better index off-task episodes than self-reported mind wandering. These findings and others examining attentional performance (see [7,17] for review) suggest that subjective reports should not be privileged as the only windows into mind wandering. Accordingly, we use both objective performance measures and subjective reports of mind wandering in the current study.

### Mindfulness Training to Strengthen Attention

The current study investigates active-duty military service-members preparing for combat. The mind’s default tendencies to wander may be a significant source of risk in the combat environment. If training to strengthen attention and curb mind wandering could be offered prior to deploying, service-members may be better able to keep their minds ‘at attention’ in a conflict zone.

One form of mental training found to be effective at improving attentional performance [18] and reducing self-reported mind wandering [19] is mindfulness training (MT). Mindfulness is described as “a mental mode characterized by attention to present moment experience without judgment, elaboration, or emotional reactivity” ([20] pg. 54). Typical MT programs offer content on how to stabilize and focus attention on one’s present moment experience, as opposed to ruminating about the past or worrying about the future. While the bulk of studies published on MT have significant time demands ranging from months of intensive daily retreat practice (e.g., [21,22]) to 8-weeks of weekly multi-hour course meetings (e.g., [21]), recent studies have found that short-duration programs are effective at reducing attentional performance lapses and self-reported mind wandering with significantly lower time demands (e.g., 7 hours, 7 weeks, [18]; 10 hours, 2 weeks, [19]). Thus, there is growing support that short-form MT programs may successfully strengthen attention.

While there is support for the effectiveness of short-form training, this effort is in its infancy with very little known about the course components critical for producing salutary effects in MT courses more generally, regardless of their time requirements. Multiple studies have suggested that MT’s beneficial effects are commensurate with the amount of time spent engaging in training exercises outside of course meetings [20,23]. Others have recently aimed to determine if biological markers and psychological health differ based on the type of training exercises (e.g., body scan, yoga, sitting meditation) in which participants chose to engage [24]. Yet, no studies have compared the effectiveness of MT programs by parsing the types of in-class activities emphasized during course meetings. In most mindfulness-based interventions, course meetings comprise guided training exercises, didactic content regarding stress and other topics, and instructor-facilitated group discussion (see [25]). In an effort to determine best practices for offering short-form MT to military cohorts preparing for deployment, the current study investigates two MT variants and compares their effects on attentional performance lapses and self-reported mind wandering. The variants emphasize *training* exercises vs. *didactic* content during 8 hours of class over 8 weeks, referred to as M8T vs. M8D, respectively.

### Building Resilience with Mindfulness Training

Resilience is the ability to maintain healthy functioning of capacities at risk for degradation over periods of intensive and persistent demands or the ability to bounce back from adversity after such intervals. Cognitive control capacities may be at risk for degradation over such intervals. Greater performance lapses and greater subjective reports of mind wandering have been reported with induced negative mood [26], dysphoria [27], craving [28], and stress ([29], but also see [30]). Recent findings also suggest that high-demand intervals may increase attentional performance lapses over time [18,31]. Might MT help build cognitive resilience by bolstering and protecting those capacities at risk of degradation during demanding and stressful periods? Specifically, might MT protect against increases in attentional performance lapses associated with mind wandering? A recent study in undergraduates investigated the putative benefits of MT to protect against performance lapses over a 7-week interval during the academic semester [18]. Compared to a no-training student group who had greater performance lapses and self-reported mind wandering on the Sustained Attention to Response Task (SART), a group receiving a 7-hour, 7-week MT course decreased their performance lapses and remained stable in self-reported mind wandering over the 7-week interval. In another study, Leonard and colleagues [31] report that high-stress cohorts of incarcerated adolescents had impaired attentional performance and increased RT variability over a 3–5 week period of incarceration. Yet, those who received a program of MT embedded within the cognitive behavioral therapy framework were significantly less impaired relative to those who received an active comparison program. These studies suggest that attentional performance lapses may increase over high-demand or high-stress intervals while MT may protect against this. Thus, the opposite of an inattentive mind may be a mindful one.

In the current study, we investigate the impact of the predeployment interval on attentional performance and self-reported mind wandering during the SART, and the putative protective effects of offering MT course variants during this interval. The military deployment cycle increases risk for psychological injury and physical harm [32]. In the several months prior to deployment to a conflict zone (the predeployment interval), service-members receive intensive training on mission-critical skills while psychologically preparing themselves to leave loved ones behind and face a potentially dangerous and unpredictable environment. While the purpose of this interval is to increase readiness to engage in their mission, several studies report impaired cognitive functioning, greater emotional disturbances, and impaired psychological health in this interval [33–35]. This corresponds with other research about the cognitive degradation associated with military stress inoculation training more generally [36–39]. Prior studies show that markers of poor psychological health (e.g., negative mood [26,40] and dysphoria [27]) correspond with greater performance lapses and greater off-task thinking during attention tasks. As such, we predict that the predeployment interval may similarly compromise attention, and that MT may protect against attentional lapses.

One prior study that investigated the protective effects of MT during the predeployment interval was described by Jha and colleagues [20]. This study aimed to determine if offering a 24-hour, 8-week MT program, called Mindfulness-based Mind Fitness Training (MMFT), might protect against degradation in working memory in predeployment Marines. Marines who participated in the MMFT course and practiced MT exercises outside of class for an average of ∼12 minutes daily maintained or improved their working memory capacity during the predeployment interval relative to Marines who practiced less outside of class or chose not to practice at all, *and* relative to Marines in the no-training control group. Thus, MMFT provided protective benefits on working memory commensurate with the amount of time spent engaging in MT exercises. Motivated by these prior results, and building on a growing literature on the inter-relationship between working memory capacity and attentional lapses associated with mind wandering (see [12,41,42]) the current project aimed to determine if offering MT to military cohorts would protect them from predicted increases in attentional lapses over the predeployment period.

While effective, the prior MMFT program was time-intensive (24 hours over 8-weeks), making its broad inclusion into military training schedules potentially challenging. An open question is whether short-form variants of MMFT could similarly protect against the predeployment interval’s deleterious effects, and if so, which components better promote successful outcomes. Two short-form MMFT variants were developed and delivered by the same team who developed the 24-hour MMFT variant [43,44]. One 8-hour MMFT variant was training-focused (M8T) and prioritized in-class instruction about and engagement in MT exercises. The other 8-hour MMFT variant was didactic-focused (M8D) and prioritized in-class instruction about the basic principles of neuroplasticity, stress, resilience, and self-regulation of the autonomic nervous system. Both variants had identical time requirements for MT exercises outside of class.

The SART was selected for the present study due to its well-documented success at indexing attentional performance lapses associated with mind wandering [17,45,46]. The version of the SART used in the present study yields a set of complementary outcome variables, including measures of 1) attentional performance errors (A’, errors of commission), 2) individual RT variability (i.e., the intraindividual coefficient of variation (ICV), and 3) subjective reports of mind wandering elicited by probes. Military participants completed the SART at two time points corresponding to the beginning (T1) and end of the MT courses (T2) during the predeployment interval.

We investigated 3 main questions. 1) Do military service-members not receiving MT (no-training control, NTC) over an 8-week high-demand predeployment interval show impaired performance at T2 relative to those experiencing 8 weeks of civilian life? Given prior results demonstrating degraded working memory capacity in predeployment military cohorts vs. civilians (CIV)[20], and the known inter-relationships between working memory and inattention [12,41], we predicted greater attentional lapses on the SART in NTC vs. CIV at T2. 2) At T2, do the MT groups outperform the NTC group, and if so, are there performance differences between the two MT groups? Given prior evidence that MT’s cognitive benefits are commensurate with time spent engaging in mindfulness exercises [20], we predicted that the M8T group, given its focus on practicing MT during class sessions, would outperform both the M8D and NTC groups at T2. 3) Does SART performance change over time in each of the four groups: CIV, NTC, M8T and M8D? We predicted the greatest vulnerability to degradation over time in NTC, and no change over time in CIV. Additionally, we predicted less SART performance degradation over time for M8T vs. M8D.

## Methods

### Participants

**Fig 1:**
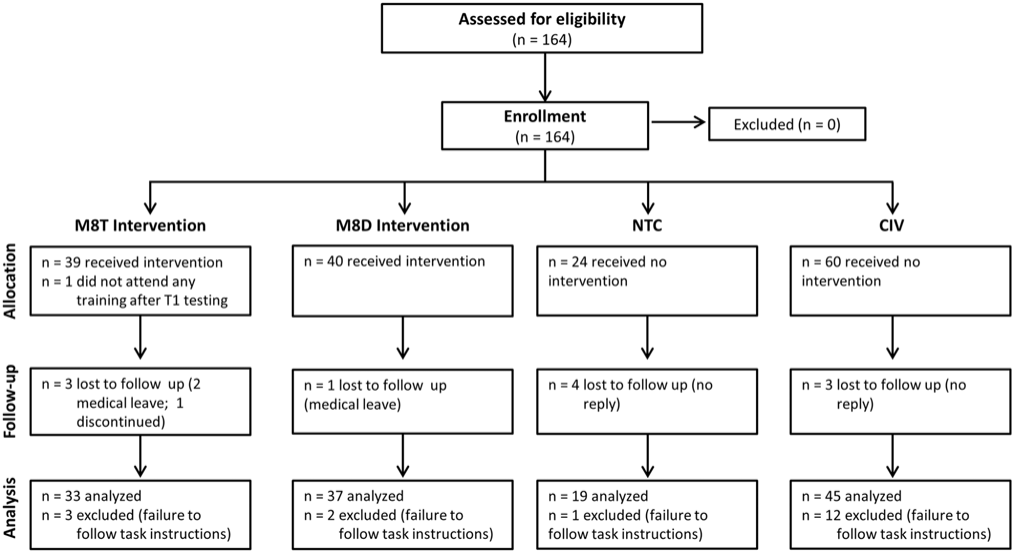
CONSORT chart for the present study demonstrating group sizes for enrollment, allocation, follow-up, and analysis.

Fig. 1 depicts the flow of all participants through each stage of the experiment. Eighty healthy active-duty U.S. Army male volunteers were recruited to participate in the MMFT training component of this project. Testing and training were conducted at Schofield Barracks, Hawaii, between 8–10 months prior to the participants’ deployment to Afghanistan. Five participants were withdrawn from the study because of scheduling conflicts during the testing sessions or their inability to engage in the training. All testing and training occurred during the soldiers’ duty day. Per Department of Defense regulations regarding soldier compensation during the duty day, they were not compensated beyond their wages for participation in this project.

In a quasi-experimental design based on available troops and command requirements that any training provided is done within the organic unit structure, soldier cohorts were randomly assigned by unit to either the 8-hour MMFT variant that emphasized didactic content (M8D) or the 8-hour MMFT variant that emphasized MT practices (M8T). This group-randomized assignment strategy is consistent with other studies in military cohorts [20,47,48]. Both groups, M8D (N = 40; age yo M = 25.8, SD = 4.5) and M8T (N = 40; age yo M = 26.7, SD = 6.2), comprised two partial combat-arms platoons with 20 soldiers from each platoon. M8D contained infantry soldiers, and M8T contained field artillery soldiers. Despite different military occupational specialties (MOS), both groups experienced similar levels of high-demand predeployment training. During their respective MMFT courses, both groups underwent MOS certification training for their respective specialties (i.e., expert infantry badge or artillery gunnery), and both groups completed similar military stress inoculation training associated with their counterinsurgency mission.

A military no-training control group (referred to as NTC) was recruited separately from the MT groups. Individuals in this group belonged to a detachment of U.S. Marine Corps Reservists preparing for deployment to Iraq (N = 24; age yo M = 27, SD = 6.1). Like the MT groups, this group received military stress inoculation training in preparation for their military advisory mission, and was tested at similar time points relative to their own deployment date as the MT groups. However, due to the challenges associated with recruiting service-members to participate in research during the high-demand predeployment interval, this group was a sample of convenience. Data from this military control group outside of the scope of the present paper and non-overlapping with the measures that are reported in the present paper have been reported elsewhere [20].

A civilian no-intervention control group (CIV), comprising individuals from the University of Miami and nearby community, was recruited through flyers to match the age of the military participants. This group comprised 60 healthy volunteers (3 female; age yo M = 20.44, SD = 3.77). Three participants withdrew from the study because of scheduling conflicts during the testing sessions; the remaining 57 participants completed both testing sessions. Civilian participants received course credit or monetary compensation. This group served to benchmark the impact of typical civilian life on our measures of interest over a similar time interval.

All participants provided informed consent in accordance with the Institutional Review Boards of the affiliated universities and with oversight from the Human Research Protections Office of the U.S. Department of Defense.

### Mindfulness Training Programs

Soldiers received two short-form variants of MMFT, a MT program created and delivered by Elizabeth A. Stanley with curriculum development support provided by John M. Schaldach [43,44]. MMFT provides a novel approach to MT designed for individuals with prior exposure to prolonged extreme stress and blends MT with concepts and skills from sensorimotor psychotherapy [49] and Somatic Experiencing [50]. A more comprehensive program description is included in Stanley [44] and Johnson and colleagues [47].

The two course variants differed in their content and delivery methods. See Table 1 for a course composition breakdown. M8T emphasized in-class practice of MT exercises and group discussion about practice; M8D emphasized lecture and group discussion of didactic information. In M8T, participants completed 4 hours of guided in-class MT exercises and in-class discussion regarding practice. M8T’s didactic framing drew from the long-form MMFT course modules linking mindfulness to concrete applications for the operational environment to motivate MT practice outside of class. Topics included how MT may strengthen attention, situational awareness, emotion regulation, and decision-making. However, M8T did not include all of the didactic information previously included in those modules (see [43]), so that the course emphasis remained on practice. Lecture and discussion in M8D related to long-form MMFT modules about basic principles of neuroplasticity, stress, resilience, and self-regulation of the autonomic nervous system, with 7 hours of instruction on these topics. The remaining hour (divided over M8D’s class sessions) introduced participants to some MT exercises and MMFT’s body-based stress resilience self-regulation exercises, and allowed for limited group discussion about practice.

**Table 1:** Composition of mindfulness training intervention.

	8-Hour Training-Focused Mindfulness-based Mind Fitness Training (MMFT) (M8T)	8-Hour Didactic-Focused Mindfulness-based Mind Fitness Training (MMFT) (M8D)
**Lecture Emphasis**	Concrete applications for mindfulness practices in the operational environment	Content about stress, resilience, neuroplasticity, and self-regulation of the autonomic nervous system
In-class time spent practicing and discussing MT exercises	50%	12.50%
In-class time spent lecturing and discussing didactic content	50%	87.50%
MT homework assigned daily	30 min.	30 min.

Both 8-hour variants in this study were delivered over 8 weeks, with the first 4 weeks comprising one 2-hour class session per week, the fifth week involving a 15-minute individual practice interview with the instructor, and the remaining three weeks involving instruction for independent practice. Both courses were interrupted by a 2-week block leave during which participants were not on post and did not receive training. The M8T group’s block leave occurred during the homework-only period (weeks 6 and 7) when they were not scheduled to meet with the instructor. The M8D group’s block leave occurred between weeks 4 and 5 (following the classes but before individual interviews). In addition to the class sessions, both groups were assigned, for the course duration, to complete 30 minutes of daily MT exercises outside of class, which could be divided into several practice periods during the day. Participants in both groups received audio CDs to guide the exercises initially, but over time they were able to do them without audio support.

Participants in the M8T and M8D groups received the same course manual, and thus participants could learn about modules they did not receive in their course through self-study. Each of the training groups also received the same audio CDs intended to guide their independent engagement in MT exercises.

**Practice Time**. Participants in both MMFT groups were asked to log their daily MT practice. Participants were encouraged to report their actual practice time as honestly as possible. They were informed that the instructor would not see these logs, which were submitted to the research team without instructor access. The mean total practice time over the 8 weeks for M8T was 178.27 minutes, SD = 277.34, and for M8D was 79.95 minutes, SD = 172.89. M8T practiced marginally more than M8D, *t*(52) = 1.755, *p* = .085. Levene’s test indicated unequal variances (*F* = 6.43, *p* <.05), so the degrees of freedom were adjusted from 68 to 52. At the end of the course, the instructor was asked to provide her assessment of the likely amount of time each participant spent engaging in MT exercises outside of class using a Likert scale (where 1 = “no practice outside of class” and 5 = “significant amount of daily practice”). Her ratings were significantly correlated with participants’ self-reported practice time for each group (M8T: *r* = .452, *p* = .007; M8D: *r* = .396, *p* = .014).

### Experimental Stimuli and Design

All participants were tested before (T1) and after (T2) an 8-week training period. They sat in a quiet room approximately 57 cm from a PC laptop display and performed the Sustained Attention to Response Task (SART; [45]). A trained experimenter proctored the session during which groups of no more than 10 participants, each at his/her own PC laptop workstation, completed the SART as well as other measures outside the scope of this report. Participants completed the SART in approximately 15–20 minutes with one self-timed break half- way through the task (after trial #330).

The SART consisted of a continuous array of single digits (0 through 9) presented visually. Each trial consisted of a digit displayed for 250 ms on a gray screen followed by a fixation cross displayed for 900 ms. Participants were instructed to withhold response (i.e., not pressing space bar) to the number 3 (target) and to respond as quickly as possible to all other numbers (non-targets). Participants could respond either during the stimulus display or during the intertrial interval (ITI). Targets were presented on 5% of trials. Trial order was pseudo-randomized so that target trials were always separated by at least one non-target trial.

**Fig 2:**
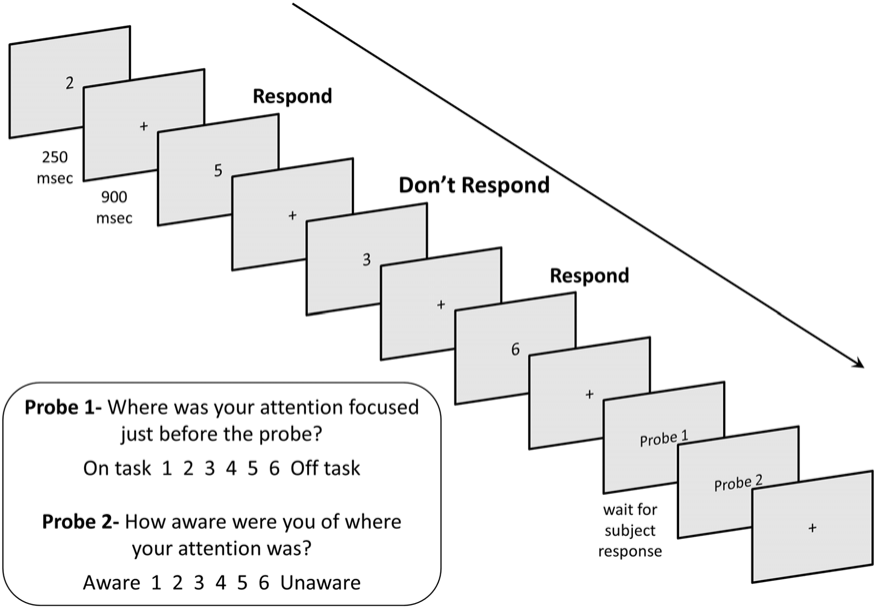
The Sustained-Attention-to-Response Task (SART). Responses were withheld from targets (the digit 3). Responses to non-targets (all other single digits) were made with the space bar. Probe 1 and 2 appeared intermittently between trials.

On occasion, two probe questions were presented in succession. The first asked, “Where was your attention focused just before the probe?” Participants responded on a 6-point Likert scale, where 1 represented “on-task” and 6 “off-task.” A second question asked, “How aware were you of where your attention was?”. Participants responded on a similar scale, where 1 represented “aware” and 6 “unaware.” The probe questions were displayed until a response was made. There were 28 probes randomly dispersed throughout all 546 trials of the task. The task began with a 163-trial practice block that included 11 probes; the results from the practice block were not included in analyses. See Fig. 2 for a sample sequence of trials and probes.

In order to provide a comprehensive picture of participants’ behavior during the SART, objective performance measures and subjective ratings of mind wandering were assessed. Objective task performance was measured by Aprime (A’) and errors of commission. A’, a nonparametric index of sensitivity [51], was calculated from “hits” (accuracy in response to targets) and “false alarms” (errors in response to non-targets) to account and correct for unequal weighting of trial types. Errors of commission (a key press in response to the target) are thought to reflect the task being performed in an automated (rather than controlled) manner, where infrequent targets go unnoticed [45].

Speed of responding was indexed as average RT and the intraindividual coefficient of variation (ICV). RT on correct non-target responses was examined with a minimum RT cutoff of 200 ms. Due to growing support that RT variability may be a valid objective performance marker of mind wandering [16,52–54], ICV was examined. A participant’s ICV is calculated by dividing the standard deviation of his/her RTs by his/her mean RT for non-target trials. Greater ICV reflects a less consistent speed of responding, and is correlated with greater self-reported mind wandering.

Subjective ratings of mind wandering were measured through participants’ average response to probe 1 and probe 2, separately. These imbedded experience-sampling probes explored the real-time (rather than retrospective) subjective experience of mind wandering.

## Results

Across all groups, 18 subjects were excluded from analyses because they did not follow task instructions (e.g., they fell asleep or did not respond to enough trials to sufficiently complete the task). The number of responses these participants recorded was more than 2 SD below the average number of responses for the group as a whole. See Fig. 1 for exclusions by group. In total, analyses were performed with 134 total subjects, including 45 participants in the CIV group, 19 in the NTC group, 33 in the M8T group, and 37 in the M8D group.

Correlations were examined to establish whether, similar to previous studies (see [16,17]), there was a significant correspondence between objective performance measures and self-reported mind wandering on the SART. As shown in Table 2, at T1 in all 134 participants, probe 1 and 2 responses were significantly positively correlated with errors of commission and ICV, and significantly negatively correlated with A’. Thus, higher degrees of self-reported “off-task” or “unaware” thinking during the SART corresponded with more errors on target trials, more variable RT, and lower signal detection. No significant relationships between either of the probes and average RT were observed. There was a significant positive correlation between responses to probe 1 and responses to probe 2, suggesting a convergent relationship between self-reports of being “off-task” and “unaware”.

**Table 2:** Correlation values for self-report and performance variables on the SART at T1.

	A’	Errors of Commission	ICV	RT	Probe 1	Probe 2
Probe 1	-.283**	.232**	.305**	.136	-	.724**
Probe 2	-.212*	.192*	.288**	.107	.724**	-

** denotes that the correlation is significant at the 0.01 level.

* denotes that the correlation is significant at the 0.05 level.

Analyses of variance (ANOVA) were performed to determine if there were group differences at T1 prior to training onset in: A’, errors of commission, average RT, ICV, and probe 1 and 2 responses. ANOVA results demonstrated significant group differences in A’ (*F*(3, 130) = 3.502, *p* = .017), probe 1 (*F*(3, 130) = 4.372, *p* = .006) and probe 2 (*F*(3, 130) = 2.837, *p* = .041). There were also marginal group differences in errors of commission (*F*(3, 130) = 2.551, *p* = .059), but no T1 differences on RT or ICV (*p* > .1). Planned contrasts revealed that at T1 CIV outperformed NTC on A’ (*t*(130) = 2.475, *p* = .015) and reported less off-task thinking on probe 1 (*t*(130) = 2.584, *p* = .011) while these two groups did not differ on probe 2 (*p* > .1). M8D outperformed M8T on A’ at T1 (*t*(130) = 2.091, *p* = .039), but did not differ from M8T on probe 1 or 2 responses (*p* > .1). M8D showed higher A’ (*t*(130) = 2.571, *p* = .011) and lower scores on probe 1 (*t*(130) = 3.583, *p* < .0005) and probe 2 (*t*(130) = 2.658, *p* = .009) when compared to NTC, while M8T had lower scores than NTC on probe 1(*t*(130) = 2.724, *p* = .007), but did not differ from NTC on A’ or probe 2 (*p >* .1).

To answer the primary question of interest regarding group differences *after* the training period and to adjust for the noted baseline differences, T1 scores were included as covariates in ANCOVA analyses of T2 scores, with group membership as a fixed factor. This ANCOVA analysis strategy was used for each measure. Significant group effects are detailed below followed by planned contrasts designed to answer specific questions about the putative costs of the predeployment interval on attentional performance and the composition of MT programs best able to protect against such costs.

There is ongoing debate regarding how to best accommodate baseline differences in outcome measures during cognitive training studies. In order to test whether our findings were tied specifically to using ANCOVA, we also examined our data with an alternative approach. A series of one-way ANOVA analyses examining the differences between T1 and T2 scores across groups for each SART outcome were also conducted, and resulted in findings that were largely consistent with the ANCOVA findings reported in detail below.

### Group-wise Differences after the Training Period

**Fig 3:**
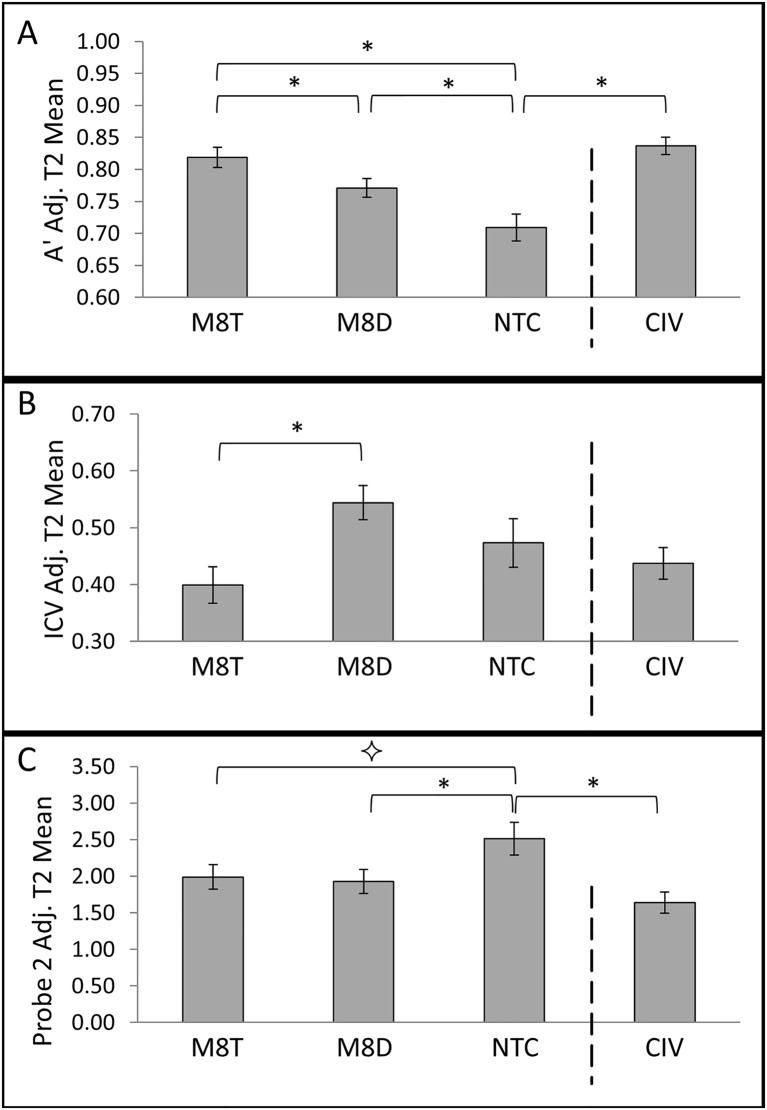
Time 2 (T2) performance in each group after adjustment for variation in Time 1 (T1) performance for (A) A’, (B) ICV, and (C) Probe 2 response. Asterisks denote a significant planned contrast at a p-value of < .05. ⟡ denotes a p-value of < .1. Error bars show standard error of the mean.

A series of ANCOVAs examining T2 with T1 as a covariate revealed significant group effects for A’ (*F*(3,129) = 10.572, *p <* .0005), errors of commission (*F*(3,129) = 4.843, *p* = .003), ICV (*F*(3,129) = 3.971, *p* = .010), and probe 2 (*F*(3,129) = 3.679, *p* = .014). Probe 1 trended towards significance (*F*(3,129) = 2.266, *p* = .084), while RT showed a non-significant group effect (*F*(3,129) = .317, *p* = .813). The significant group effects from the ANCOVAs were examined in more detail through a series of planned contrasts. Fig. 3 depicts the adjusted T2 means from each group for A’, ICV, and probe 2.

**Comparison of Civilian Control Group vs. Military Control Group**. First, the military (NTC) and civilian (CIV) control groups were compared to test whether SART measures differ after enduring a high-demand 8-week predeployment interval relative to a similar time period of civilian life. Indeed, A’ scores were lower in NTC (adj. mean = .701) vs. CIV (adj. mean = .837, *p* < .0005, 95% CI [.078, .178]), and there were more errors of commission in NTC (adj. mean = 71%) vs. CIV (adj. mean = 53%, *p* = .001, 95% CI [.071, .285]). ICV did not differ between NTC (adj. mean = .473) and CIV (adj. mean = .437, *p* = .137, 95% CI [-.64, .137]). For probe 2 (where numerically higher responses correspond to less awareness of one’s own attention), NTC (adj. mean = 2.514) reported higher scores than CIV (adj. mean = 1.641, *p* = .001, 95% CI [.348, 1.398]).

**Comparison of Military Groups**. M8T and M8D were separately compared to NTC. A’ scores in M8T (adj. mean = .819) were significantly higher than those reported in NTC (adj. mean = .709, *p* < .0005, 95% CI [.509, .162]) and M8T had fewer errors of commission (adj. mean = 56%) vs. NTC (adj. mean = 71%, *p* = .010, 95% CI [.036, .258,]). ICV did not differ between M8T (adj. mean = .399) and NTC (adj. mean = .473, *p* = .166, 95% CI [-.031, .181] while responses to probe 2 were marginally lower in the M8T group (adj. mean = 1.989) vs. NTC (adj. mean = 2.514, *p* = .064, 95% CI [-.030, 1.081]).

A’ scores were significantly higher in M8D (adj. mean = .771) than NTC (adj. mean = .709, *p* = .018, 95% CI [.011, .114]). Yet these groups did not differ on errors of commission (M8D adj. mean = 64%, NTC adj. mean = 71%, *p* = .250, 95% CI [-.046, .175]) or ICV (M8D adj. mean = .544, NTC adj. mean = .473, *p* = .178, 95% CI [-.174, .033]). However, probe 2 responses were significantly lower in M8D (adj. mean = 1.927) vs. NTC (adj. mean = 2.514, *p* = .038, 95% CI [.034, 1.142]).

Next, M8T and M8D were directly compared to each other to determine if M8T’s focus on practicing MT exercises led to fewer attentional lapses and more self-reported awareness relative to the M8D group. M8T (adj. mean = .819) had significantly higher A’ scores than M8D (adj. mean = .771, *p* = .029, 95% CI [.005, .091]). Errors of commission trended towards significance between M8T (adj. mean = 56%) and M8D (adj. mean = 64%, *p* = .083, 95% CI [-.011, .175]), with fewer errors for M8T vs. M8D. ICV was lower in M8T (adj. mean = .399) than M8D (adj. mean = .544, *p* = .001, 95% CI [.057, .233]). Responses to probe 2 did not significantly differ between groups (M8T adj. mean = 1.989, M8T adj. mean = 1.927, *p* = .789, 95% CI [-.523, .398]).

Thus, collectively, the groupwise differences in multiple objective measures reviewed above suggest that M8T (vs. M8D) was more effective at protecting against attentional performance lapses and instability at T2. Of note, A’ significantly differed in each of our groupwise comparisons of interest. Specifically, within the military groups, A’ was greatest for M8T followed by M8D and lowest for NTC. To determine if these groupwise differences may be related to within-group degradation in performance over the predeployment interval (from T1 to T2), further analysis of A’ was conducted separately for each group.

### Change Over Time

**Fig 4:**
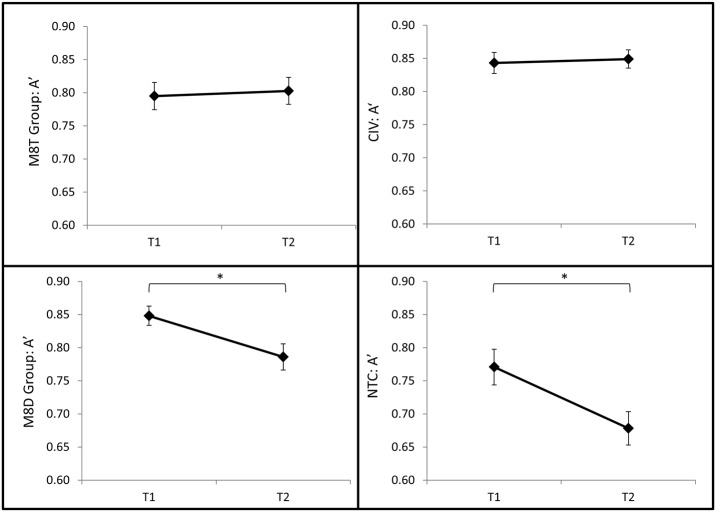
A’ in each group from T1 to T2. While the M8T and CIV groups did not change over time, the M8D and NTC groups degraded from T1 to T2. Asterisks denote a significant planed contrast at an alpha of .05. Error bars show standard error of the mean.

We examined whether attentional performance lapses (measured by A’) change over time for each group by running a series of paired t-tests for each group separately. While no change over time was seen in CIV (*t*(44) = .532, *p* = .603) and M8T (*t*(32) = .381, *p* = .706), NTC (*t*(18) = 3.364, *p* = .003) and M8D (*t*(36) = 3.999, *p* < .0005) significantly degraded from T1 to T2 (Fig. 4).

## Discussion

In the current study we investigated the impact of short-form MT on SART performance in predeployment military cohorts. We asked three main questions. 1) Are attentional lapses greater in those who have endured a high-demand predeployment interval relative to a similar time period of civilian life? At T2, the NTC group had lower A’ scores and more errors of commission relative to CIV. In addition, relative to CIV, NTC reported being significantly less ‘aware’, as indexed by their T2 probe 2 scores. Thus, objective task performance and subjective reports expose the adverse cognitive effects of the predeployment interval. 2) Does MT protect against these deleterious effects of the predeployment interval? At T2, both MT groups had higher A’ scores and self-reported being significantly (M8D) or marginally (M8T) more ‘aware’ of their attention compared to NTC. In addition, a direct comparison of the two MT groups revealed that M8T had higher A’ and lower ICV relative to M8D, suggesting fewer attentional lapses and greater attentional stability in the M8T group. Yet, the MT groups did not differ on probe 2 scores at T2. As such, while both MT groups report being more subjectively ‘aware’ than NTC at T2, the two MT courses may not *differentially* strengthen meta-awareness. 3) Does MT promote cognitive resilience by protecting against degradation in attention *over time*? To gain a more complete picture of the cognitive costs of enduring a high-demand interval such as preparing for military deployment vs. a similar time period of civilian life, changes in A’ over time were examined separately for each group. As predicted, CIV A’ scores remained stable over time while the NTC group’s scores significantly declined from T1 to T2. Like NTC, M8D also significantly degraded over time, suggesting that participating in this MT course did not sufficiently protect against performance costs over time. In contrast, M8T’s A’ scores remained stable from T1 to T2. These results suggest that attentional performance, which degrades over the predeployment interval, is better protected by M8T vs. M8D. These results suggest that MT focused on in-class training exercises more so than on in-class didactic instruction may promote cognitive resilience by protecting attentional capacities put at risk by high-demand intervals.

Prior research confirms that the SART is a stable task [45], and this was supported herein by the CIV group’s performance pattern, which did not change over time. In contrast, NTC performed more poorly at T2 vs T1. In addition, at T2 they had lower performance and self-reported being less ‘aware’ than CIV. Might the high-demand interval endured by NTC, without compensatory MT to complement their military training, have put them at risk for depletion of attentional resources? During the predeployment period, military service-members typically learn new operational tasks, engage in field training exercises, and must psychologically prepare themselves to leave loved ones behind and face uncertain and potentially life threatening situations [33,34]. Attentional resources available to address the myriad of intensive and persistent cognitive and emotional challenges may be continuously taxed over time.

A resource depletion framework of attention (see [55]) has been proposed in a growing literature on the specificity and nonunitary nature of executive control processes. Many studies testing this framework use laboratory tasks to tax specific attentional processes over a few to several minutes and interpret performance lapses in targeted processes as markers of fatigue from overuse. In line with prior laboratory-based research, the NTC group’s SART performance lapses suggest that the persistent real-world demands they experienced in the predeployment interval may have depleted their attention over the course of a few months. While not the focus of the current study, future research should aim to better capture the types of processes most vulnerable to being taxed during the predeployment interval. In clinical contexts, well-developed task batteries, which fractionate executive control processes across tasks, are used to probe specific vulnerabilities in patient populations [56]. A parallel effort to examine cognitive vulnerabilities that may emerge as service-members prepare for deployment is warranted (see [57]).

The central aim of the current project was to determine if MT might protect against degradation in SART performance suffered by those enduring high-demand intervals, such as preparing for military deployment. Comparison of NTC to the MT groups helps clarify the impact of the high-demand interval on the SART versus the putative benefits of offering MT in that interval. M8D’s task performance was higher and they self-reported being more ‘aware’ than NTC at T2. Yet, similar to NTC, M8D’s A’ scores degraded from T1–T2. A direct comparison of M8D to M8T confirms that the training-focused emphasis of M8T may offer superior protection from degradation of attentional resources relative to the didactic emphasis of M8D. Indeed, M8T outperformed M8D at T2, and M8T’s A’ scores did not degrade from T1–T2.

While NTC self-reported being near significantly or significantly less ‘aware’ at T2 relative to M8T and M8D, the MT groups did not differ from each other in their Probe 2 scores. Probe 1, which indexed self-reports of being on-task vs. off-task did not significantly differ across the four groups, and was not further explored. Thus, while significantly correlated with the objective performance metrics on the SART, probe 1 and 2 did not provide conclusive evidence for differential effectiveness between M8T and M8D. There are several reasons why these subjective reports may not have differed between MT groups. One possibility is that the didactic content common to both MMFT programs was sufficient to result in comparable benefits in meta-awareness relative to NTC. Alternatively, the minimum amount of MT practice time achieved by both groups may have similarly benefitted subjective awareness beyond NTC. A third possibility is that responses to probes provide a crude and imperfect assessment of mind wandering. This assessment may have lacked the precision to distinguish M8T from M8D even if there was less mind wandering happening in the M8T group. Indeed, prior research suggests that ICV may better capture mind wandering than self-report metrics [16]. In line with this, M8T’s ICV scores were significantly lower than M8D’s. Nonetheless, more research about the degree of overlap between objective and subjective reports of mind wandering is necessary before any strong claims can be made.

In contrast to the subjective measures, the results from the objective performance measures are more straightforward. Why might the M8T group have outperformed M8D? As has been previously suggested, the SART has many elements that may index “near transfer” from the cognitive processes exercised by MT [18]. In theory, both MT groups may have benefitted SART performance because of similarities between the SART and the type of MT exercises that are included in both short-form variants. For example, during many exercises, participants are instructed to focus on one target object of attention (such as body sensations or sounds) and maintain this focus over the practice period. When they notice the mind wandering, they are instructed to redirect attention toward the target object. Likewise, during the SART, participants must attend to a continuous string of digits over the course of the task while noticing and limiting the mind’s tendency to wander. Accordingly, the processes trained during MT exercises, such as deploying and sustaining attention, could be applied towards SART performance. Yet, only the M8T and not the M8D group maintained stable SART performance over time. This pattern suggests that mindfulness training delivered via a *training-focused* (vs. didactic-focused) course may result in more effective transfer of skill from the MT exercises to the SART. Although both groups were taught the same MT exercises, only M8T’s emphasis allowed for ample access to instructor-led MT practice, opportunities to discuss and receive feedback about MT exercises, and greater time spent practicing MT exercises in class. In addition, the M8T group reported greater (marginally significant) time spent out-of-class engaging in MT exercises. As such, it is unclear if M8T’s better SART performance (relative to M8D) was due to greater in-class practice time or cumulative practice time.

Various SART performance measures have recently been proposed to index distinct aspects of attention. Errors of commission, for example, have been suggested to indicate that the SART is being completed in an automated manner rather than one reflecting controlled attention [45]. Peaks in reaction time variability are thought to reflect the degree of attentional disengagement from the task [58]. Similarly, errors of omission, a factor used to calculate A’, are proposed to index complete inattention to and disengagement from the task at hand [46]. M8T and M8D differed in errors of commission, ICV, and A’, either significantly or near-significantly at T2. These findings suggest the two MT programs may have differed in the promotion of controlled attention as well as protection against inattention.

While the comparisons between the military groups do provide insights into the attentional costs of the predeployment interval and possible protective benefits of MT, it is important to note the limitations in the selection of units and the randomization procedures that were required to feasibly conduct this project in an active-duty context. While all three military groups were recruited into the study during their predeployment interval as they prepared for their respective deployments, there are many differences to note. The two groups receiving MT were US Army soldiers preparing for a counterinsurgency combat deployment to Afghanistan whereas the NTC group was a US Marine Reserve unit preparing for a military advisory mission deployment to Iraq. Thus, unlike the two MT groups, the NTC was not part of the same parent unit and command climate, not preparing for the same deployment mission or theater, not from the same military service or service component (Marine reservists called to active duty for deployment vs. active-duty Army soldiers), and likely did not share the same military service culture. In short, the two MT groups were substantially more similar to each other than to the NTC group. Nonetheless, all three military groups included troops from combat arms military occupational specialties, preparing for deployment for missions involving population-centric operations in theaters with the possibility of active combat. The NTC group was included as a convenience sample and was previously part of a separate project (see [20]). NTC was included instead of recruiting and involving a better-matched active-duty Army combat arms unit, because of the scarcity of access to such units in the current deployment context. Indeed, a non-combat arms Army no-training group from the parent unit of the MT groups was part of a larger research project that is outside the scope of this paper. This group was less similar to the two MT groups (in terms of gender mix, MOS, predeployment training regimen, and mission during deployment) than the Marine combat arms NTC here. In sum, it is important to keep in mind that baseline differences in military cohorts included as part of this report may have contributed to the group-wise differences that were observed.

We acknowledge that this study has other design limitations as well, and suggest that many are tied to the challenges associated with studying active-duty military service-members. In this population, research participation is secondary to the primary aim of being prepared for military missions. Our research effort functioned within their respective organizational structures and was bounded by training schedule constraints. MT randomization occurred by group, with participants assigned to M8T or M8D based on their platoon assignment. While training groups were matched as best as could be accommodated (age, gender, predeployment training regimen, expected mission during deployment, and point in the deployment cycle), there were baseline differences in the SART at T1, and these differences guided our analysis strategy of treating T1 scores as covariates. Individual differences in SART performance in healthy, young adults have been previously documented [4]. Still, it is imperative that future studies use larger sample sizes to comprehensively explore a full spectrum of demographic variables, cognitive, affective, personality, and motivational factors that may contribute to variability in SART performance observable as group-wise differences. Yet, it is possible that this method may not avoid baseline differences. Another strategy would be to engage in blocked matching of specific measures at T1 or multivariate matching before individual randomization.

The results of the present study should be replicated in larger cohorts of troops, with a study design including random assignment at the individual level, and cognitive performance effects measured longitudinally over the entire deployment cycle. Yet, we acknowledge that such designs, while theoretically appropriate and experimentally ideal, may not be practically appropriate or feasible in active-duty contexts due to troops’ military training schedules. Thus, research in this context aims to best accommodate high research standards while accepting that this is secondary to the military mission. Future studies should also examine biological markers of stress (see [47]) in order to investigate the relationship between cognitive performance, mindfulness training, and stress over the high-demand predeployment interval.

Many prior MT studies have been criticized for inadequate designs, comparing MT to waitlist no-training controls and failing to compare against active control training. In studies that have carefully created well-matched comparisons to MT programs, the control program aims to match for instructor expertise, enthusiasm, and personality, as well as psychosocial support, in and out of class time demands, and participant expectations of benefits (see [59]). The current study was designed with these principles in mind. The same trainer offered M8T and M8D, and course content for each was drawn from longer-form MMFT developed by this trainer [43,44] and found to be effective [20,43,47]. Thus, many of the limitations regarding appropriate controls for which numerous prior studies of MT have been criticized were addressed in this design. Accordingly, better performance of the M8T vs. M8D group at T2 cannot be attributed to instructor-related differences, differences in psychosocial support, or expectations.

Prior studies of longer-form MMFT, comprising all components of both short-form MMFT variants investigated herein and additional components, have shown efficacy in benefitting cognitive markers (i.e., working memory capacity, 24-hour MMFT course [20]), as well as neural and biomarkers of self-regulation and resilience (20-hour MMFT course, [47]). Given scheduling challenges in predeployment training calendars, this study sought to determine if shorter-form MT variants are effective. The current results did advance understanding of which of the course components included in longer-form MMFT are best able to promote salutary effects with short-form MMFT in the predeployment interval. Although time constraints may motivate greater consideration of offering short-form MT to high-stress cohorts during high-demand training intervals, future studies must address whether the beneficial effects of decreased attentional lapses endure over time as very little is known about the enduring effects from studies of short-form MT. One final question is the availability of trainers qualified to offer MT to high-stress groups, and over the entirety of the deployment cycle. Train-the-trainer methods may be a route forward, as has been implemented for other military resilience programs [60].

In sum, the current results suggest that protracted periods of high demand experienced during military service-members’ predeployment training may increase attentional lapses. However, MT programs akin to M8T, which emphasize engaging in MT exercises, may protect against associated performance costs. Short-form MT should be further considered as a method to bolster resilience of cognitive capacities at risk for degradation in high-stress environments. Thus, MT may be a route by which to keep a soldier’s mind ‘at attention’.

## Supporting Information

S1 Dataset Spreadsheet containing dataset for the present manuscript. (XLSX)
